# A Decadal Shift in Time Use Among Chinese Older Adults: A Comparative Analysis of the 2008 and 2018 Time Use Surveys

**DOI:** 10.1093/geroni/igaf122.2343

**Published:** 2025-12-31

**Authors:** Jing Ye, Hongyan Yang

**Affiliations:** Wuhan University, Wuhan, Hubei, China (People’s Republic); Wuhan University, Wuhan, Hubei, China (People’s Republic)

## Abstract

China is undergoing rapid population aging, with 22% of its population aged 60 and above in 2024 and an increasing dependency ratio. In response to mounting labor shortages and pension pressures, the government initiated a progressive extension of the retirement age in September 2024. Against this backdrop, this study investigates the evolving time-use patterns of older adults (aged 60 and above) by analyzing data from the 2008 and 2018 China Time Use Surveys. Findings reveal that, for paid labor, the employment duration of older adults increased significantly (with workdays rising by 0.28 hours and rest days by 0.30 hours), while weekday household production participation rate declined from 36% to 24%. This reduction in total labor time underscores tensions with delayed retirement policies. Regarding unpaid labor, although overall housework participation fell from 79% to 70% on workdays, time devoted to childcare increased by 0.12 hours, with persistent gender disparities—women engaged in 1.53 more hours of housework than men. Leisure and socialization activities shifted notably, with internet use growing by 16% on mobile devices and leisure time expanding by 1.37 hours on workdays, though traditional activities such as television viewing and fitness remained dominant. Additionally, self-care practices improved, as indicated by increased time spent on sleep and meals. These results suggest a transition toward lighter labor burdens and enriched leisure activities among older adults, while also reinforcing intergenerational support despite enduring traditional gender roles. The study offers empirical insights to inform policies on retirement reform and caregiving infrastructure in aging societies.

